# Supplementary material for: Chromothripsis during telomere crisis is independent of NHEJ, and consistent with a replicative origin
Source: Genome Res. 2019 May;29(5):737–49. doi: 10.1101/gr.240705.118 (PMC6499312; doi:10.1101/gr.240705.118)
Supplement: Supplemental Material [file supp_gr.240705.118_Supplemental_file_1.zip › contigs/annotated_contigs/DB53/contig.10.DB53_length_2061_mean_cov_25.3551673945.docx]

**DB53_length_2061_mean_cov_25.3551673945**

```
ATAAAGGGTCCCAAATTTTCAGATTCTGAAGTTATTTTGAAAACTTTTAAATATACTCTCGTCTCCCTGCTTTTCATCAGTCCATTGAC
 >chr8:80743125-80743506 - E=1e-216
CAATCACTCTGGTTATCAATGCACCTATTTCCAGAAATTCTTCTACCCTAATTTTCTGATGTTCAGTGTTTACTGTGAGCATAGAAGAC

CAATATAGACACACACTTACACGTGTGTGCATGCAGATGTGCATTAGAACTGTGTGTAGAGAGGTTTGAGGAGGTACCTGAGAGTTTCC

TATGAGTAAATGGAATATGTTATGAGTTCTCTGTTTTGGTTTTCCAGAGACTGCTTTTGTTAAATTTTTTGCTCATTTTAGGAATAGAA

AGTTGCAAGACAAATGAGTTTAATA|TGCT|CCTGTTCTGAGTAAGAATACATTCAAGTCTTATAGTTTCATGGCTTAAAGAAATCCAA
                              >chr8:88456778-88456965 - E=4e-100
GGTTGATTCAATTTATGTGTATAAAGTCTGAAGGTAACTGGTCCAATTCACCGATGTAGTTCAGAAAGTGGGCTCTTGCTGTCTTTATG

GTACAACATCTCTTTTTGTGCTACATGTTAACAATATGGT|GTAAGTGA|TGGCAACTGGCTCATGGAGAAGCAGCTGCTGGTCCCTCA
                                                 >chr8:81610164-81610367 + E=9e-110
CTAGAGTTTCCAGTCGGTGCTTTGGCAAAGATACTGCTCATTACATAATGAGTCTTGGCCAGACTGTCCACAAATAGAAAAGCCTAGAG

TGCTTGTTTGGATTCTACGTTTAAGAGTTTATGCTTTCAATACTCACTCTTGGAGCACTAGGGCTGTGCTAGAC|A|CACTAGAAATGT
                                                                           >chr17:68800
CAAGTAGTGTTGGAGGAGAGACA|AGA|GTTTCACTCTGTTGCCCAGGCTGGAGTGCAATGGTGCGATCTCGGTTCAGTGCAGCCTCTG
498-68800537 - E=3e-11  >chr8:81120982-81121211 - E=2e-125 p=0e+00
CCTCCCAGGTTCCAGTGATTCTCCTGCCTCAGCCTCCCGAGTAGCTGGGATTACAGGCATGCGCCACCACACTCGGCTAATTTTTTTGT

ATGTTTAGTAGAGATGGGGTTTCATCATGTTGGCCAGGCTGGTCTCGAACTCCTGACTTCAAATCATCTAC|CCACC|CTGATAATTTT
                                                                        >chr17:68550119
TTGTATTTTTAGTAGAGATGGGGTTTGGCCATGTTGGGCAGGCTGGTCTCGAACTCCTGACCTCAGGTAAGCCACCTGCCTAGGCCTCC
-68550412 - E=7e-164
CAAAGTGCTGGGATTACAGGCGTGAGCCACACAATTTTGACTGAAAAATTAAAGGTGAGTAAACTTTCCCCTTGATAGGTGCAAAAACT

GTTGTGCACAAATCAGCTGCAGACAAGAACAGAGCTTTCAATGAAAATTTTAAACAAATAGAATCAAGATCCTGAAGTATTCTTCAAAG

AATTGTAACA|AA|TTTGTGAGCAATATTTATTACATATACATTCAAGCTGGGTTTTGTTCTGTTTTTTTTTGAAGAGGATATATTAAT
             >chr8:78552342-78552425 + E=3e-26
AACT|CCTGGGCTCA|AGTTTCTGCTTCTAGGCTTTTGGCTGGGCAGCTGCTGCCTTCCAACAGGAAATTTATTAAAATGCATTTCAAT
               >chr8:79142093-79142205 - E=4e-55
CCTACAAAAGTCTATCTGGCATTGGTTATTAATTTTGCT|CTGTGGATTG|TGTATTTAGTGAAAGAGAAACAAACAAACAAAA|ACG|
                                                  >chr8:78542328-78542361 - E=1e-07
GTTTCAATTCAGTAAGAGAATTAAAGAAATATAAGAAAATAAAACTCTATTAGGGATGTGATTATGTCAGATTAATTCTTTGTTTTCAA
>chr8:85465838-85466316 - E=3e-270
GGCATTTTAAAAAGCATTTTGTATTCTAAAGTATTTGAAAATAGTTATGTGTATGGAAAGATGATTCATCTTAACAGCAAATGTTTGTG

TAACACTTAAATTTTCTGCTTCTACTACTTAAAGTCACTTGGCTAATAATATTTAAATATTAACATAGATATACACGTAAGAAATAAAA

TAGACAGTATAGGCAAAATACATTAATTTTCTTAAAGGACCACTGCCACCTGTAGCTTGATTTTACTTTGGATATTTAATAAATTTTCA

CTCCTTTTTTTTTCCAGGCACAATAAGAAAATGTTACTTTAGAGGTTACAAAATAATTTAATAGATATTCTTTTAGAAATACTTCCAAT

TCTTAACTGTGCTATTTTTACCTTGAAACAAT
```
